# Isolation and characterisation of nasoseptal cartilage stem/progenitor cells and their role in the chondrogenic niche

**DOI:** 10.1186/s13287-020-01663-1

**Published:** 2020-05-14

**Authors:** Zita M. Jessop, Ayesha Al-Sabah, Irina N. Simoes, Stephanie E. A. Burnell, Ina Laura Pieper, Catherine A. Thornton, Iain S. Whitaker

**Affiliations:** 1 Reconstructive Surgery and Regenerative Medicine Research Group, Institute of Life Sciences, Swansea University Medical School, Swansea, SA2 8PP UK; 2 Welsh Centre for Burns and Plastic Surgery, Morriston Hospital, Swansea, SA6 6NL UK; 3 Calon Cardio-Technology Ltd, Institute of Life Sciences, Swansea, SA2 8PP UK

**Keywords:** Tissue-specific stem cell, Cartilage tissue engineering, Chondroprogenitor, Cartilage stem cell, Nasoseptal cartilage, Stem cell niche, Chondrogenic niche

## Abstract

**Background:**

Since cartilage-derived stem/progenitor cells (CSPCs) were first identified in articular cartilage using differential adhesion to fibronectin, their self-renewal capacity and niche-specific lineage preference for chondrogenesis have propelled their application for cartilage tissue engineering. In many adult tissues, stem/progenitor cells are recognised to be involved in tissue homeostasis. However, the role of nasoseptal CSPCs has not yet been elucidated. Our aim was to isolate and characterise nasoseptal CSPCs alongside nasoseptal chondrocyte populations and determine chondrogenic capacity.

**Methods:**

Here, we isolated nasoseptal CSPCs using differential adhesion to fibronectin and assessed their colony forming efficiency, proliferation kinetics, karyotype and trilineage potential. CSPCs were characterised alongside non-fibronectin-adherent nasoseptal chondrocytes (DNCs) and cartilage-derived cells (CDCs, a heterogenous combination of DNCs and CSPCs) by assessing differences in gene expression profiles using PCR Stem Cell Array, immunophenotype using flow cytometry and chondrogencity using RT-PCR and histology.

**Results:**

CSPCs were clonogenic with increased gene expression of the neuroectodermal markers NCAM1 and N-Cadherin, as well as Cyclins D1 and D2, compared to DNCs. All three cell populations expressed recognised mesenchymal stem cell surface markers (CD29, CD44, CD73, CD90), yet only CSPCs and CDCs showed multilineage differentiation potential. CDC populations expressed significantly higher levels of type 2 collagen and bone morphogenetic protein 2 genes, with greater cartilage extracellular matrix secretion. When DNCs were cultured in isolation, there was reduced chondrogenicity and higher expression of type 1 collagen, stromal cell-derived factor 1 (SDF-1), CD73 and CD90, recognised markers of a fibroblast-like phenotype.

**Conclusions:**

Fibronectin-adherent CSPCs demonstrate a unique gene expression profile compared to non-fibronectin-adherent DNCs. DNCs cultured in isolation, without CSPCs, express fibroblastic phenotype with reduced chondrogenicity. Mixed populations of stem/progenitor cells and chondrocytes were required for optimal chondrogenesis, suggesting that CSPCs may be required to retain phenotypic stability and chondrogenic potential of DNCs. Crosstalk between DNCs and CSPCs is proposed based on SDF-1 signalling.

## Introduction

The ability to successfully tissue engineer cartilage would have a significant impact on the ability to reconstruct cartilaginous defects and thereby restore function. Contemporary cartilage tissue engineered implants, often using unrelated adult stem cell sources, do not produce stable, physiologically relevant cartilage [1, 2]. Diseases with great health economic and physical burdens such as osteoarthritis, propagated by the low self-repair properties of cartilage, have stimulated research into articular cartilage tissue engineering [3–5]. There has been a wide range of alloplastic, autologous and composite attempts to produce cartilage implants, which have been associated with a variety of complications including inflammation, resorption, extrusion, infection and migration [6–8]. Cell integration in the form of chondrocytes has been used to reduce complication rates with mixed results [6, 9–12]. The use of stem cells in cartilage tissue engineering was a major development in the field. Although mesenchymal stem cells (MSCs) from unrelated sources including the bone marrow, adipose tissue and skeletal muscle demonstrate chondrogenic potential, the majority of studies have shown that these MSCs often lead to fibrotic and calcified cartilage with poor mechanical properties and low physiological relevance [10, 13–16].

Since they were first identified in the articular cartilage using differential adhesion to fibronectin [17–20], cartilage-derived stem/progenitor cells (CSPCs) have been added to the repertoire for cartilage tissue engineering [21–24]. CPSCs have been characterised by convention of the International Society of Cellular Therapy (ISCT) based on the demonstration of plastic adherence, multipotency and positivity for stem-cell-related surface markers [17, 19, 25, 26]. As a clonogenic, renewable cell source derived from the specific tissue that requires replacement, CSPCs are of interest due to their niche-specific lineage preference for chondrogenesis [27–29].

In the present study, we isolated cells from human nasoseptal cartilage due to relative ease of access for biopsy and favourable donor site morbidity versus articular cartilage [30]. This donor site offers several other advantages including the ability of nasal chondrocytes to adapt to heterotopic transplantation to articular sites and potent chondrogenic potential which is hyaline specific [31–33]. Although previous studies have suggested a role for isolated CSPCs for cartilage tissue engineering, none have characterised them alongside chondrocytes or in mixed populations to investigate their possible role in phenotype modulation and chondrogenesis. As seen in vivo, the tissue microenvironment and stem cell niche plays a significant role in contributing to the phenotypic stability of cells and chondrogenesis [34]. The aim of this study was therefore to fully characterise heterogenous cell populations in nasoseptal cartilage. Cartilage stem/progenitor cells (CSPCs) were isolated using differential adhesion to fibronectin and characterised alongside differentiated nasoseptal chondrocytes (DNCs) and cartilage-derived cells (CDCs, a combination of DNCs and CSPCs). We demonstrate that heterogenous cell populations have greater chondrogenic potential than the use of stem/progenitor cells alone, indicating important crosstalk that may contribute to phenotypic stability.

## Materials and methods

### Isolation of human nasoseptal cartilage stem/progenitor cells (CSPCs)

Adult human nasoseptal cartilage was obtained from healthy donors undergoing septorhinoplasty after obtaining informed consent at Singleton and Morriston Hospitals, Swansea, UK. All procedures were approved by the ABM University Health Board (IRAS ID 99202). Surface fibrous tissue was removed, and the remaining blood was washed away using Dulbecco’s modified Eagle medium (DMEM, Thermofisher Scientific, Waltham, MA, USA) prior to mincing the cartilage into ~ 1-mm^3^ pieces. Sequential tissue digestion was performed with gentle agitation using 2 mg/ml pronase (Roche, Basel, Switzerland) solution for 40 min and 2.4 mg/ml collagenase (Sigma-Aldrich, St. Louis, MO, USA) for 16–18 h, all at 37 °C [35]. The enzymatic solutions were prepared using DMEM supplemented with 1% penicillin-streptomycin solution (PS, Thermofisher Scientific), 1 mM d-glucose solution (Thermofisher Scientific) and 0.1% minimum essential medium (MEM) non-essential amino acids (NEAA, Thermofisher Scientific). Digested tissue was filtered through a 40-μm cell strainer (VWR, Radnor, PA, USA) and centrifuged for 5 min at 500*g*. Cells were re-suspended in chondro-medium (CM, DMEM supplemented with 10% foetal bovine serum (FBS, Thermofisher Scientific), 1% PS, 1 mM d-glucose and 0.1% MEM NEAA) and plated (1) in culture grade plastic surface (i.e. CDCs) or (2) in fibronectin-coated surface using a previously reported protocol [18, 20, 29, 36]. Briefly, 6-well plates (Sigma-Aldrich) were coated with 10 mg/ml fibronectin (Sigma-Aldrich) in Dulbecco’s phosphate-buffered saline (DPBS, pH 7.4, Thermofisher Scientific) containing 0.5 mM magnesium chloride (MgCl_2_) and 0.9 mM calcium chloride (CaCl_2_). Cells were seeded at low density (2000 cells/well) in CM and incubated for 20 min in standard culture conditions (i.e. 5% CO_2_ and 37 °C). Non-adherent cells were collected and seeded on new non-coated 6-well plates (i.e. DNCs). Fresh CM was added to the fibronectin-adherent CSPCs. Once confluent, all three cell populations were passaged on culture grade plastic under standard culture conditions and the same cell density, with medium changes every 2–3 days. Cells were trypsinyzed (0.05% trypsin-EDTA, Thermofisher Scientific) for 5 min at 37 °C, centrifuged at 587*g* for 5 min, resuspended in fresh CM and re-seeded at 6.7 × 10^3^ cells/cm^2^. Cells were kept in culture under standard conditions up to passage 13 (P13).

### Growth kinetics of CDCs, DNCs and CSPCs

Short-term cell proliferation was determined using the RTCA iCELLigence™ system (ACEA Biosciences, San Diego, CA, USA). P2 cells were seeded in 8-well E-plates at 10,000 cells/well and CM under standard culture conditions. Cell attachment and proliferation were monitored in real time based on cellular impedance. Wells containing CM only were used as negative controls. The cell index (CI) is a function of the cell number and ratio of cells at different time intervals; CI = 0 when there is no cell adhesion. The CI in a RTCA system is the result of the impedance induced by adherent cells to the electron flow. CI is calculated as follows: CI = (impedance at time point n-impedance in the absence of cells)/nominal impedance value. Measurements for CI were taken every minute for the first 2 h and then every hour for 24 h for all three cell populations (CDC, DNC and CSPC).

Long-term proliferative capacity in culture was determined by measuring cumulative population doublings (PD) at each cell passage [37]. Cell growth was determined between P1 and P13 by direct cell counts using trypan blue exclusion method. PDs were calculated using the formula below where *N* represents cells harvested/cells seeded and used to plot growth curves.

$$ \mathrm{PD}=\log 10(N)/\log 10(2) $$

### Colony forming efficiency (CFE) assay of CSPCs

CSPCs were seeded at P0 at 200 cells/cm^2^ and cultured in standard culture conditions with medium changes every 2–3 days. After 14 days, fibroblast-like colony forming units (CFU-F) were counted under an optical microscope. A colony was defined as > 32 cells [18]. Colony forming efficiency (CFE) was calculated as a percentage of CFU-F from the initial number of cells seeded [36, 38] and used as a predictor for the proportion of CSPCs within the original nasoseptal cartilage cell population.

### Trilineage differentiation of CDCs, DNCs and CSPCs

For osteogenic and adipogenic differentiation, cells were seeded (osteogenic, 20,000 cells/well and adipogenic, 40,000 cells/well) onto 12-well plates (Sigma-Aldrich) and cultured in CM for 3 days under standard culture conditions. The medium was then changed to either StemPro™ osteogenesis differentiation medium (Thermofisher Scientific) or StemPro™ adipogenesis differentiation medium (Thermofisher Scientific). Cultures were maintained for 21 days under standard culture conditions with medium changes every 2–3 days. For chondrogenic differentiation, 500,000 cells were resuspended in 1.5 ml Eppendorf in StemPro™ chondrogenesis differentiation medium (Thermofisher Scientific) and centrifuged for 5 min at 783*g*. Pellets were cultured for 21 days under standard culture conditions with medium changes every 2–3 days. Negative control samples of each cell type were maintained in CM. After 21 days, cells were fixed in 4% paraformaldehyde (PFA, Alfa Aesar, Haverhill, MA, USA) for 30 min. Osteogenesis and adipogenesis were confirmed using 2% Alizarin Red S and 0.3% Oil Red O, respectively. A 1% Alcian blue solution prepared in 0.1 N hydrochloric acid (HCl) was used to confirm the presence of glycosaminoglycans (GAGs) in chondrogenic differentiation. Stains were visualised using phase-contrast AmScope MD35 microscope (AmScope, Irvine, CA, USA).

### Histological staining

Cells were fixed in 4% PFA for 30 min and washed in phosphate-buffered saline (PBS, Thermofisher Scientific). Cells were stained with 1% Alcian blue (TCS Biosciences, Buckingham, UK) solution for 15 min and washed with water, while others were stained with 0.1% toluidine blue (TCS Biosciences) solution in water for 3 min following wash with water. For safranin-O stain, cells were exposed to 0.1% fast green (TCS Biosciences) solution for 10 min, immersed in 1% acetic acid for 10 s (Sigma-Aldrich), stained with 0.1% safranin-O (TCS Biosciences) for 20 min and washed with water. All stains were visualised using phase-contrast AmScope MD35 (AmScope).

### Flow cytometry

CDCs, DNCs and CSPCs from P1 and P8 were immunophenotypically characterised using flow cytometry and a panel of mouse anti-human monoclonal antibodies against CD29, CD34, CD44, CD45, CD56, CD73 and CD90 (all from Biolegend, Supplementary Table S1). P1 and P8 cells were used to assess phenotype at both early and late passages. Unstained cell populations were used as controls. A minimum of 10,000 events were collected for each sample, and data was acquired using a Novocyte® flow cytometer (ACEA Biosciences) and analysed by FlowJo® software (FlowJo, LLC, Ashland, OR, USA). The geometric mean fluorescence intensity (MFI) for each cell surface marker was used as quantitative measure of expression relative to the unstained controls to allow the level of expression to be compared between the three cell populations. Percentage of cells expressing each cell surface marker was used as a further quantitative measure to allow comparisons between the three cell populations.

### RNA extraction, quantitative real-time PCR and PCR array

Total RNA was extracted using Trizol (Thermofisher Scientific) and chloroform (Sigma-Aldrich) and purified using RNeasy Mini Kit (Qiagen, Hilden, Germany) according to the manufacturer’s instructions. RNA concentration was determined using NanoDrop (Thermofisher Scientific) and converted into cDNA using Superscript IV reverse transcriptase (Thermofisher Scientific) following manufacturer’s protocol. Quantitative PCR (qPCR) was completed using Human Stem Cell RT^2^ Profiler™ PCR Array (Qiagen, Supplementary Figure S1) according to the manufacturer’s protocol and using iCycler Real-Time PCR system (Bio-rad, Hercules, CA, USA). Gene expression was quantified using GeneGlobe Data Analysis Center software (Qiagen) and normalised to *RPLP0*, using CDCs as a control. A volcano plot was generated to compare CSPCs and DNCs.

### Cytogenetic analysis

Karyotyping was performed through a collaboration with the Institute of Medical Genetics from Cardiff and Vale University Health Board. Cells from passage 1 and passage 4 from 5 human donors were used to investigate whether prolonged expansion during in vitro culture causes any gross karyotype changes that may warrant further genotoxicology studies, with a view to autologous cartilage-derived cells being utilised for tissue engineering in the future. Passage 4 was chosen because it provides enough cells for seeding into 3D scaffolds and there are several reports in the literature that CSPCs can maintain capacity to be reprogrammed into chondrocytes even after passage 4 [39, 40]. Briefly, cells at 70% confluence were exposed overnight and under standard culture conditions to BioWhittaker® Amniochrome II medium (Lonza, Basel, Switzerland) containing 37.5 μg/ml of 5′-Bromo-2′-deoxyuridine (BrdU, Sigma-Aldrich) and 0.6 μg/ml Colcemid (Invitrogen, Carlsbad, CA, USA). Cultures were washed with PBS, trypsinised using trypsin/EDTA solution (Life Technologies, Carlsbad, CA, USA) for 2 min and resuspended in a 1:10 FBS (Thermofisher Scientific) and sterile water solution. Cell suspensions were fixed by adding 3:1 methanol-acetic acid fixative (VWR, BDH Chemicals) followed by centrifugation at 487*g* for 10 min (performed twice) and 2:1 methanol-acetic acid followed by another centrifugation. Pellets were resuspended in 2:1 methanol-acetic acid fixative, spread on slides and dried at a relative humidity of 50%. For Giemsa banding (GTG-banding), slides (aged 3–5 days at room temperature) were placed in trypsin solution for 5–10 s, rinsed in 3 changes of normal saline and stained in 10–20% RA Lamb Giemsa stain (Thermofisher Scientific) in phosphate buffer pH 6.8 (VWR, BDH Chemicals) for 1.5 min. After rinsing in 3 changes of phosphate buffer pH 6.8, slides were dried and mounted in Entellan mountant (Merck, Kenilworth, NJ, USA).

### Statistical analysis

Statistical data are represented as means ± standard error of the mean (SEM) unless otherwise indicated. One-way ANOVA was applied to calculate *p* values. Statistical differences between groups for the same experimental set were determined using Tukey post hoc test. Statistical analysis was performed using Minitab® 18 (Minitab, Inc., State College, PA, USA). A *p* value ≤ 0.05 was considered significant.

## Results

### CSPCs show increased expression of *CCND1*, *CCND2*, *NCAM1* and *CDH2* genes compared to DNCs

**Fig. 1:**
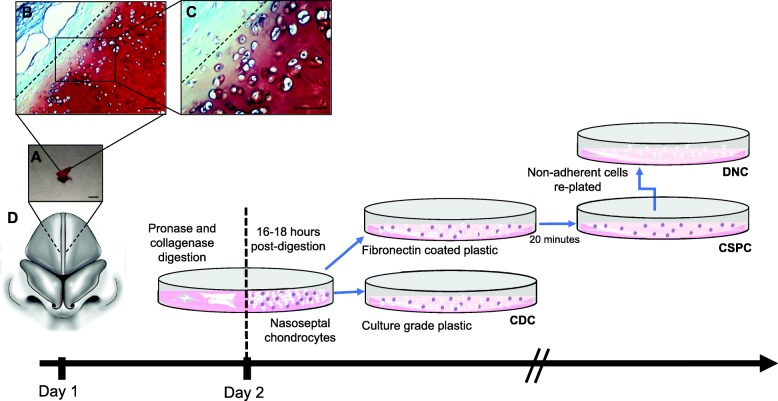
Isolation of nasoseptal cartilage-derived cells. **a** Gross morphology of nasoseptal cartilage taken from patients undergoing septorhinoplasty. Scale bar, 4 mm. **b**, **c** Safranin-O staining of nasoseptal cartilage indicating the fibrous tissue removed (dashed line) prior to enzymatic digestion. Scale bars, 100 μm. **d** Schematics of the isolation of CDC, DNC and CSPC populations. Briefly, at day 1, the tissue is subjected to enzymatic digestion for 16–18 h. CDCs are seeded in culture grade plastic while the remaining cells are separated based on fibronectin adherence: after 20 min on fibronectin, the non-adherent population (DNCs) is transferred to a separate culture plate. Once confluent all three cell populations were passaged on culture grade plastic under standard culture conditions and the same cell seeding density

CSPCs were isolated using differential adhesion to fibronectin from fifteen patient donors following routine septorhinoplasties (Fig. 1). Cells which were not adhered to fibronectin were referred to as DNCs, and the original cell population containing both populations were referred to as CDCs. Nasoseptal cartilage samples (292 ± 124 mg) yielded 11,022 cells/mg of tissue with over 90% viability.

**Fig. 2:**
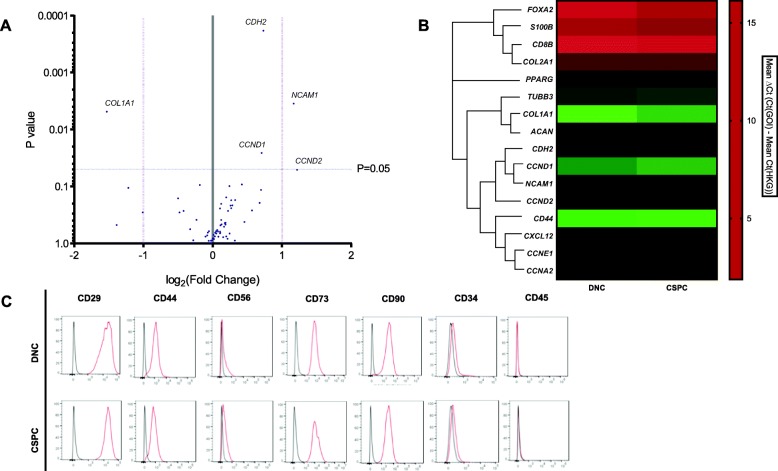
Genetic and flow cytometric profiles of CSPC and DNC cell populations. **a** Volcano plot of PCR array depicting the average of log2 (fold change) and *p* value of the mRNA expression level of stem cell markers in CSPCs versus DNCs (*n* = 7). **b** Heat map and hierarchical clustering dendrogram of the average 2-ΔCt values of co-regulated genes that were significant upregulated or downregulated in at least three biological samples in the CSPC and DNC populations. **c** Flow cytometry characterisation of cell surface markers on CSPCs and DNCs

Genetic and flow cytometric profiles of the fibronectin-adherent (CSPC) and non-adherent (DNC) cell populations were examined at P1. There is an increase in the expression of neural cell adhesion molecule 1 (*NCAM1/CD56*) by 2.2-fold (*p* value < 0.01) and N-cadherin (*CDH2*) by 1.7-fold (*p* value 0.001) in CSPC versus DNC populations. Proliferative genes, cyclin D1 (*CCND1*) and cyclin D2 (*CCND2*), were also shown to be significantly upregulated in CSPCs compared to DNCs (*p* < 0.05) (Fig. 2a). Additionally, a decrease in type 1 collagen (*COL1A1)* expression (*p* value < 0.01) was observed (Fig. 2a). A hierarchical clustering dendrogram analysis of a heat map of mean 2-ΔCt values for selected genes from PCR array revealed that the significantly upregulated genes *NCAM1*, *CDH2*, *CCND1* and *CCND2* in CSPCs cluster together (Fig. 2b). Both CSPC and DNC populations demonstrated cell surface expression of CD56, recognised MSC markers (CD29, CD44, CD73, CD90) and a lack of haematopoietic markers (CD34, CD45) (Fig. 2c).

### CSPCs are a slow proliferating clonogenic subpopulation from human nasoseptal cartilage

**Fig. 3:**
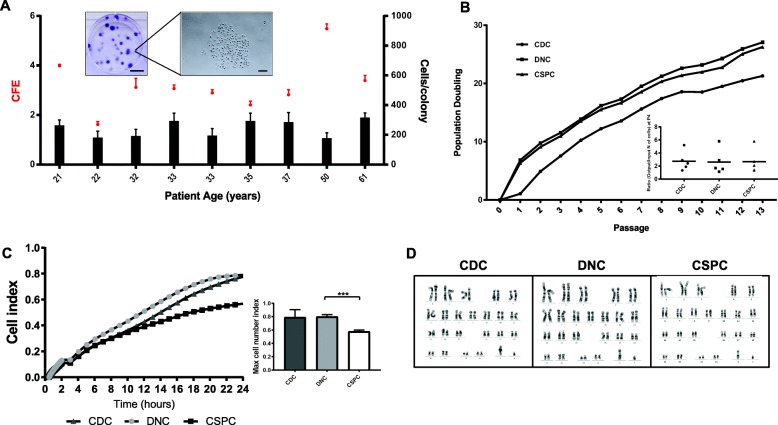
Characterisation of proliferating CDC, DNC and CSPC populations. **a** Colony forming efficiency (CFE; left axis—red) of CSPCs from nine donors and number of cells/colony (right axis—black) after 14 days in culture. Upper panel insert: representative crystal violet staining of CSPC colonies. Scale bars = 10 mm and 100 μm (left to right). **b** Population doublings (PD) of CDC, DNC and CSPC cultures along cell passages (P1–P13). Inset represents patient variability (*n* = 5 at passage 4). **c** Changes in cell index over time according to RTCA iCELLigence™ assay (*n* = 3) with bar graphs to demonstrate maximum cell index reached after 24 h in culture, using RTCA iCELLigence™ assay (*n* = 3). Results as mean ± SEM, ***p < 0.001. **d** G-band karyotype analysis of CDCs, DNCs and CSPCs at P4 (46XY) representative analysis from 5 human donors analysed for changes at P1 vs P4

**Fig. 4:**
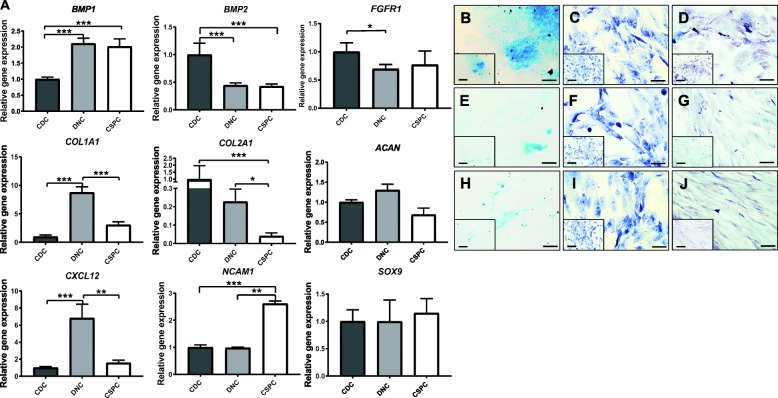
Chondrogenic potential of CDC, DNC and CSPC populations. **a** Relative gene expression of chondrogenic-, proliferative- and progenitor-related genes in CDCs, DNCs and CSPCs. Expression levels were normalised to the level of *RPLP0*, and relative expression was calculated against CDCs. *CCND2*, cell cycle cyclin D2; *BMP2*, bone morphogenetic protein 2; *COL1A1*, type 1 collagen; *COL2A1*, type 2 collagen; *ACAN*, aggrecan; *CXCL12,* C-X-C motif chemokine 12; *NCAM1*, neural cell adhesion molecule 1; *SOX9*, sex-determining region Y box 9. Results as mean ± SEM from 6 independent experiments (*n* = 6) in technical triplicates, *p < 0.05, **p < 0.01 and ***p < 0.001. **b**–**j** Histology staining of **b**–**d** CDCs, **e**–**g** DNCs and **h**–**j** CSPCs at P2 using **b**, **e** and **h** Alcian blue; **c**, **f** and **i** Safranin-O; and **d**, **g** and **j** toluidine blue. Images are representative from 3 independent experiments (*n* = 3). Scale bars, 100 μm

CSPCs were demonstrated to be clonogenic with an average CFE of 3.2 ± 0.15 (Fig. 3a), suggesting 3.2% of cells in the original isolated nasoseptal cell population are CSPCs. All three cell populations showed comparable long-term growth curves and achieved at least 20 population doublings over 13 passages (over 200 days in culture). The slope of the CDC growth curve appeared to reduce after passage 9 potentially indicating senescence (Fig. 3b). The impedance-based proliferation assay demonstrated that the DNC population had greater proliferative capacity by reaching a significantly higher maximal cell index compared to the slower-cycling CSPC population (0.80 ± 0.05 and 0.58 ± 0.04, respectively, *p* < 0.001, Fig. 3c). Karyotype analysis indicated no chromosomal abnormalities for all three cell populations after four passages in culture (P1 vs P4) and thereby feasibility for in vitro expansion towards cartilage tissue engineering applications (Fig. 3d). As part of the genes evaluated by PCR array, *CCND2* was shown to be significantly downregulated in DNCs (8.3-fold, *p* < 0.001) and CSPCs (5.3-fold, *p* < 0.001) with respect to CDCs (Fig. 4a).

### CDCs demonstrate greater chondrogenicity

The chondrogenic potential of the three populations was investigated at the gene and protein level. PCR arrays showed CDCs expressed significantly higher levels of type 2 collagen (*COL2A1*) than DNC and CSPC populations (Fig. 4a). Interestingly, *COL2A1* was significantly downregulated in CSPC subpopulation by 24-fold (*p* < 0.01) with respect to CDCs and 5.5-fold (*p* < 0.05) with respect to DNCs (Fig. 4a). Bone morphogenetic protein-2 (*BMP2*) expression has exhibited significant downregulation in DNCs and CSPCs by 2.2-fold and 2.3-fold respectively compared to CDCs (*p* < 0.001, Fig. 4a). However, aggrecan (*ACAN*) and *SOX9* mRNA expression is observed to be expressed at similar levels in all cell types (Fig. 4a). In contrast, at the protein level, CDCs are observed to have enhanced glycosaminoglycan secretion as evident by the intense Alcian blue (Fig. 4b, e and h) and safranin O (Fig. 4c, f and i) staining in comparison to DNC and CSPC populations. While the toluidine blue staining is of similar intensity in all three cell populations, the distribution of the staining is sparser in the DNCs and CSPCs when compared to the CDC population (Fig. 4d,g and j).

### CDCs and CSPCs demonstrate multilineage potential in vitro

**Fig. 5:**
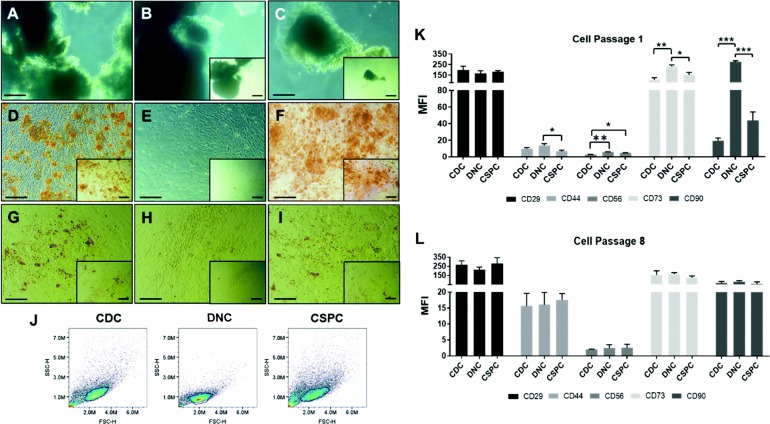
Nasoseptal CSPCs are multipotent and positive for MSCs markers. **a**–**i** Multilineage differentiation ability of CDCs (**a**, **d**, **g**), DNCs (**b**, **e**, **h**) and CSPCs (**c**, **f**, **i**) into **a**–**c** chondrogenic, **d**–**f** osteogenic and **g**–**i** adipogenic lineages at P2. Images are representative from 3 independent experiments (*n* = 3). Scale bars, 100 μm. **j**–**l** Flow cytometry characterisation of CDCs, DNCs and CSPCs. **j** Dot blots representative of cell populations from P1. **k**, **l** Mean fluorescence intensity (MFI) calculated for P1 and P8 cells, respectively. Results as mean ± SEM from three biological repeats (*n* = 3), *p < 0.05, **p < 0.01 and ***p < 0.001

The ability of chondrocyte populations to commit to trilineage differentiation was compared in vitro (Fig. 5a**–**i). CDC and CSPC populations demonstrated trilineage commitment whereas DNCs have limited lineage plasticity. DNC cultures possessed no osteogenic and poor adipogenic potential (Fig. 5e, h) as evident by limited staining with Alizarin red and Oil Red O respectively. All three subpopulations (CDCs, DNCs and CSPCs) stained positively for Alcian blue following chondrogenic differentiation in pellet culture (Fig. 5a**–**c).

### DNCs commit to a fibroblastic lineage in the absence of CSPCs

Early passage (P1) nasoseptal cells were further evaluated to determine if the cultured cell populations present distinct immunophenotype profiles (Fig. 5j, k and Table 1). CD29 expression was shown to be at a similar level across all cell types, showing no significant differences (> 165 MFI, Fig. 5k and > 97%, Table 1). CD44 was significantly downregulated in CSPCs in comparison to DNCs with a MFI fold decrease of 2.1 (*p* < 0.05, Fig. 5k). Although CSPCs present lower CD44 MFI than CDCs (6.81 and 9.92 MFI, respectively, *p* > 0.05, Fig. 5k), there is higher expression percentage in CSPCs (83.9% and 70.4%, respectively, *p* > 0.05, Table 1), indicating that this population has more cells expressing CD44, but those expressing it have fewer receptors present. The expression of CD56 is significantly higher in DNCs and CSPCs when compared to CDCs (2.1 and 1.7 MFI fold increase, respectively, *p* < 0.05, Fig. 5k and 51.3% and 47.9% increase, respectively, *p* < 0.0001, Table 1).

**Table 1:** Percentage of expression of different cell surface markers in CDC, DNC and CSPC populations at P1. Results as mean ± SEM (*n* = 3). Significant *p* values are indicated; -- represents non-significant values

	Percentage of expression (%)			***p*** values		
	CDC	DNC	CSPC	CDC vs DNC	CDC vs CSPC	DNC vs CSPC
**CD29**	97.86 ± 0.92	99.31 ± 0.24	99.19 ± 0.27	--	--	--
**CD44**	70.39 ± 7.46	96.43 ± 1.15	83.91 ± 2.95	0.026	--	0.017
**CD56**	3.67 ± 1.90	55.02 ± 6.37	51.61 ± 10.82	0.001	0.012	--
**CD73**	99.01 ± 0.27	99.64 ± 0.06	99.47 ± 0.16	--	--	--
**CD90**	93.10 ± 0.47	99.71 ± 0.02	99.27 ± 0.13	0.000	0.000	0.031

DNCs showed a significantly higher MFI for CD73 and CD90 when compared to the two other cell populations (2.1- and 1.5-fold increase for CD73 when compared to CDCs and CSPCs, respectively, *p* < 0.05, and 14.2- and 6.3-fold increase for CD90 when compared to CDCs and CSPCs, respectively, *p* < 0.001, Fig. 5k). CD73 showed over 99% expression in all cell populations, with DNCs showing higher expression, which was not found to be significant (*p* > 0.05, Table 1). On the other hand, CD90 expression was significantly increased in DNCs when compared to the other two cell populations (6.6% increase compared to CDCs, *p* < 0.0001, and around 0.4% increase compared to CSPCs, *p* < 0.05, Table 1).

Other markers indicative of fibroblastic lineage were found to be elevated in the DNC population including *COL1A1* (8.8-fold with respect to CDCs *p* < 0.001 and 5-fold with respect to CSPCs *p* < 0.001, Fig. 4a) and C-X-C motif chemokine 12 (*CXCL12*) (6.8-fold with respect to CDCs *p* < 0.001 and 5.3-fold in comparison to CSPCs *p* < 0.01, Fig. 4a). MSC markers have been investigated at a later passage (P8) to determine the effects of culturing DNC and CSPC populations separately; however, there were no significant changes across cell populations indicating that these immunophenotypic differences are not maintained after prolonged culture (Fig. 5l).

## Discussion

To date, no optimal cell source has been identified for cartilage tissue engineering purposes. The aneural and avascular nature of cartilage tissue should theoretically make it an easier tissue to replicate compared to other specialised tissue types with more heterogenous functions. However, recapitulating native, functional cartilage remains a challenge [2] and has therefore been the focus for tissue engineering research for many years [41, 42]. Chondrocytes are well documented to undergo fibroblastic differentiation after prolonged expansion in traditional monolayer culture with loss of chondrogenicity [43, 44]. MSCs tend to produce calcified or hypertrophic cartilage [10, 13, 14]. Cartilage-specific stem cells have shown diminished capacity to retain optimal chondrogenic potential when cultured in isolation [45, 46], exhibiting low chondrogenic markers and failing to form a functional matrix in vivo [47–50]. Nevertheless, it is well established that almost all connective tissues contain tissue-specific stem/progenitor cells which have a significant role in tissue homeostasis and maintenance [51, 52]. Recent studies also speculate that tissue-specific stem/progenitor cells help limit dedifferentiation and maintain phenotypic stability in other tissue types by modulating the local environment [52].

In this study, we successfully isolated CSPCs from human nasoseptal cartilage and characterised them alongside DNC and CDCs. Trilineage differentiation analysis indicated that the multilineage potential observed in CDCs was due to the presence of the CSPC subpopulation, evident from the absence of osteogenic and adipogenic staining in the DNC population. DNCs showed higher proliferation rates than CSPCs, which is in line with the literature addressing the slow proliferation rates of stem/progenitors cultured separately [53]. Interestingly, Cyclin D1 and Cyclin D2 gene expression was greater in CSPCs despite their lower proliferation rates. The differentiation fate of stem cells is tightly tied to cell cycle regulation and cyclins D1 and 2, whose activity is required for cell cycle G1/S transition, has been associated with stem/progenitor cells from a variety of tissue types and can control cell fate and differentiation through transcriptional networks and epigenetic modifiers [54, 55].

All cell populations were positive for recognised MSC markers including CD29, CD44, CD73 and CD90 while being negative for the haematopoietic markers CD34 and CD45. To our knowledge, this is the first study to report CD56/*NCAM1* expression in all subpopulations of adult chondrocytes which may be attributed to the previously reported neuroectodermal developmental origin of nasoseptal cartilage in the literature [32]. NCAM1 has been shown to be expressed in embryonic chondroprogenitor cells and mediates cell-cell adhesion during the early stages of cartilage development [56, 57]. However, NCAM1 was not previously thought to be expressed in adult chondrocytes, where the primary interactions were believed to be integrin mediated cell-matrix contacts [58]. Accumulating evidence suggests that biomechanical signals originating from cell-cell adhesion are critical for stem cell lineage specification, and studies have suggested CD56/NCAM1 as a putative marker of connective tissue stem/progenitor cells [59–61], but further work is required to determine whether it is a marker of stemness or dedifferentiation in nasoseptal chondrocytes. It may be that in the mixed CDC populations chondrocytes remain in a more differentiated state hence lower MFI and percentage CD56/NCAM1 expression, in keeping with its role during early condensation stage of cartilage development [53].

Our findings suggest that CSPCs may act as a supporter cell to stabilise the chondrogenic phenotype of DNCs. When cultured in isolation, DNCs and CSPCs demonstrated significantly reduced gene expression of chondrogenic markers such as type 2 collagen as well as proteoglycan histological staining compared to the mixed CDC population. DNCs and CPSCs also showed significant downregulation of *BMP2*, which is recognised in the literature for its requirement in chondrogenic matrix synthesis [62]. Interestingly, DNCs adopt a fibroblastic phenotype in the absence of CSPCs, evidenced by increased expression of *COL1A1* and cell surface expression of CD73 and CD90, which are known fibroblastic markers [63, 64] whose expression increases in a time-dependent manner [65]. The expression of the fibroblastic marker, *CXCL12*, which encodes stromal cell-derived factor 1 (SDF-1), was also observed to be significantly increased in the DNC population following a similar trend to *COL1A1* and CD90 [66]. This is of interest as SDF-1 is a chemokine that acts on surrounding cells and has multiple roles including promoting proliferation and progenitor differentiation [67, 68].

**Fig. 6:**
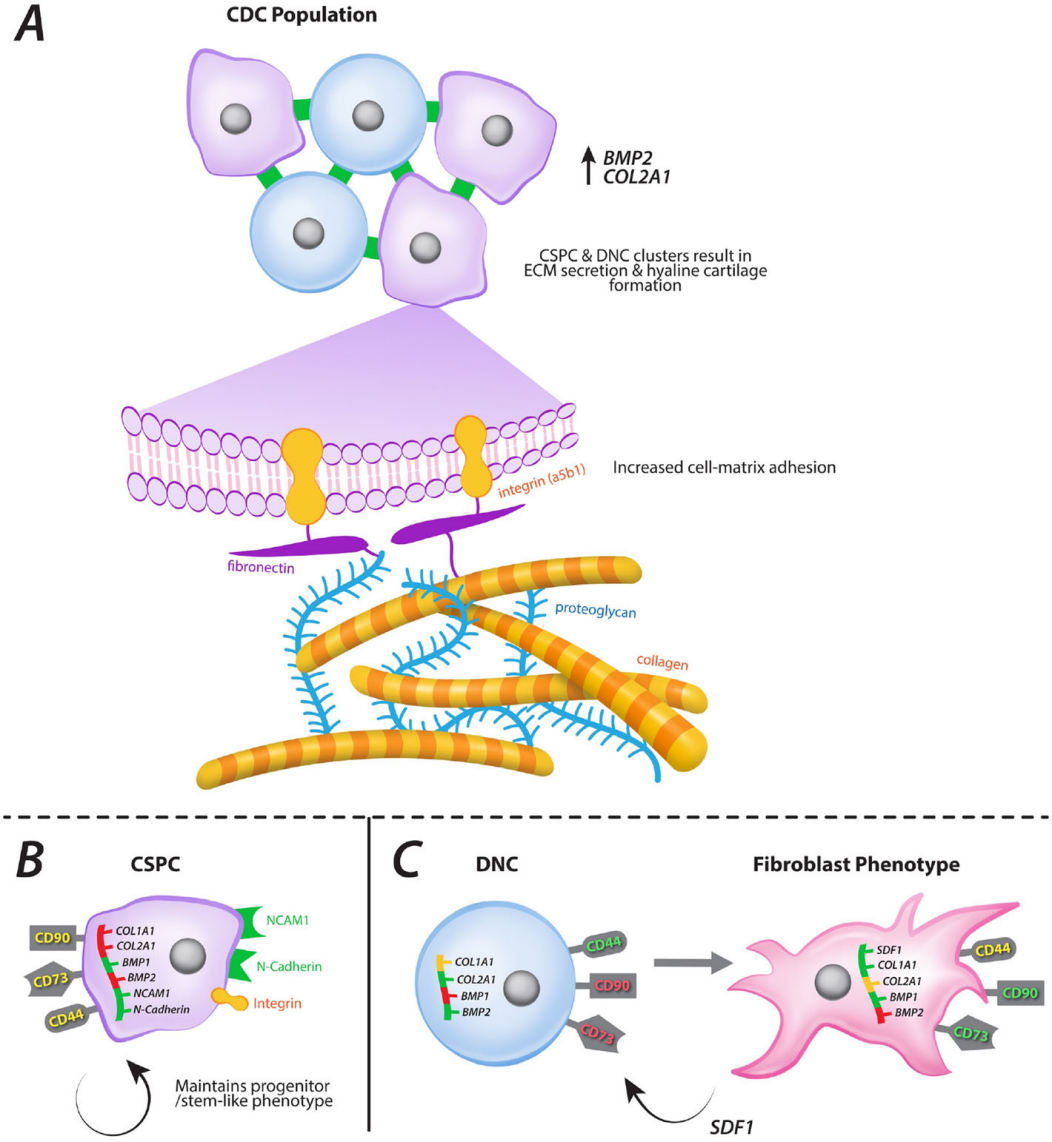
Proposed mechanism of the support role of CSPCs in the nasoseptal cartilage cell niche. **a** CDC population comprises DNCs and CSPCs which reside together in the cartilage niche, expressing high levels of BMP2, COL2 and CCND2 and secreting normal hyaline cartilage extracellular matrix (ECM). The crosstalk between the two cell populations may be based on SDF-1: DNCs secrete SDF-1 which induces CSPC differentiation into DNCs, supporting the maintenance of a chondrogenic environment. When **b** DNCs and **c** CSPCs are separated and cultured individually, their expression profile and phenotype are altered. **c** CSPCs alone tend to maintain their progenitor-like phenotype, showing increased expression of CD56/NCAM. These cells are clonogenic and hold multilineage differentiation potential as demonstrated here. **b** When DNCs are deprived of CSPCs, these will change from a chondrogenic to a fibroblast-like phenotype, expressing high levels of COL1, SDF-1, CD90 and CD73. Consequently, DNCs alone will have decreased chondrogenicity and poor secretion of cartilage ECM. CCND2, cell cycle cyclin D2; BMP2, bone morphogenetic protein 2; COL1A1, type 1 collagen; COL2A1, type 2 collagen; SDF-1, stromal cell-derived factor 1; NCAM, neural cell adhesion molecule. Created using BioRender©

Our study indicates that the co-existence of CSPC and DNC populations produces the optimal chondrogenic phenotype in vitro and the putative mechanism for this is proposed in Fig. 6. Collectively, the results indicate a potential regulatory role for DNCs in recruitment of CSPCs through SDF-1, which may in turn either differentiate into chondrocytes or release extracellular signalling factors to promote DNC chondrogenic phenotype stability, thereby contributing to the greater chondrogenicity observed in the CDC population [69, 70] (Fig. 6). DNC-CSPC interaction may be essential, to maintain chondrogenic gene expression and extracellular matrix synthesis. These findings are supported by a previous in vivo study, where mixed populations, i.e. CDCs, maintained a more chondrogenic phenotype when injected intramuscularly in mice when compared to an isolated population of chondroprogenitors [48]. When cultured individually, the CSPC and DNC populations have distinct *CCND1*, *CCND2*, *COL1A1*, *COL2A1*, *CXCL12*, *NCAM1* and *CDH2* gene expression profiles, indicating either progenitor status or fibroblastic lineage respectively [59, 60] but lacking optimal chondrogenicity.

## Conclusions

This study provides insight into the role of nasoseptal CSPCs in the in vitro chondrogenic niche and maintenance of phenotypic stability of nasoseptal chondrocytes through influence on dedifferentiation. Mixed populations of stem/progenitor cells and chondrocytes were required for optimal chondrogenesis. Further work will elucidate the molecular mechanisms underlying this phenomenon, enriching the translational potential of tissue engineered cartilage and associated cell-based therapies**.**

## Supplementary information

**Additional file 1 : Supplementary Table S1.** Detailed information on fluorescent dye and excitation and emission wavelengths of the antibodies used for flow cytometry. AF, Alexa Fluor; APC, allophycocyanin; BV, brilliant violet; FITC, fluorescein isothiocyanate; PE, phycoerythrin; PerCP, peridinin-chlorophyll protein complex.

**Additional file 2 : Supplementary Figure S1.** Detailed information on the contents of the RT^2^ Profiler™ Human Stem Cell PCR Array as supplied by Qiagen, including gene description and position in array.

## Data Availability

The datasets generated during the current study are available in the figshare repository which can be downloaded from https://figshare.com/s/f2fb966577d8c655d171. All other datasets used and/or analysed during the current study are available from the corresponding author on reasonable request.

## Abbreviations

ACAN: Aggrecan gene
BMP2: Bone morphogenetic protein-2
CCND2: Cyclin D2
CCND1: Cyclin D1
CDC: Cartilage-derived cell
CFE: Colony forming efficiency
CFU-F: Fibroblast-like colony forming units
CM: Chondro-medium
COL1A1: Type 1 collagen
COL2A1: Type 2 collagen
CSPC: Cartilage-derived stem/progenitor cell
CXCL12: C-X-C motif chemokine 12 or stromal cell-derived factor 1
DMEM: Dulbecco’s modified Eagle medium
DNC: Differentiated nasoseptal chondrocyte
FBS: Foetal bovine serum
GAG: Glycosaminoglycan
ISCT: International Society of Cellular Therapy
MEM: Minimum essential medium
MSC: Mesenchymal stem cell
NCAM1: Neural cell adhesion molecule 1 or CD56
NEAA: Non-essential amino acids
PFA: Paraformaldehyde
RPLP0: Ribosomal protein lateral stalk subunit P0
SDF-1: Stromal cell-derived factor 1
SOX9: Sex-determining region Y box 9
