# Supplementary figures and images for: Isolation and characterisation of nasoseptal cartilage stem/progenitor cells and their role in the chondrogenic niche
Source: Stem Cell Res Ther. 2020 May 14;11:177. doi: 10.1186/s13287-020-01663-1 (PMC7222513; doi:10.1186/s13287-020-01663-1)

## Slide 1
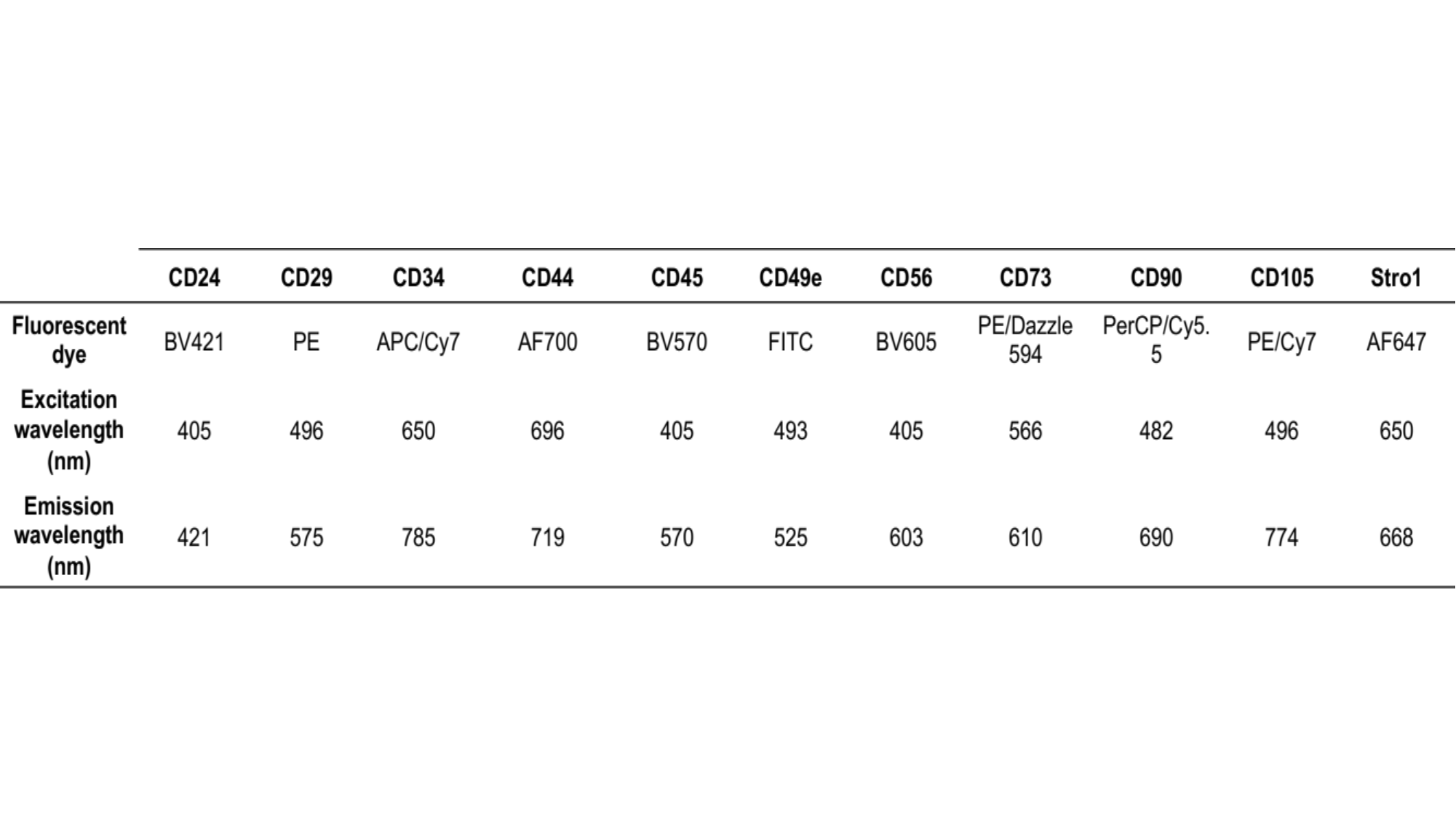

Supplement: Supplementary file 1 — Additional file 1 : Supplementary Table S1. Detailed information on fluorescent dye and excitation and emission wavelengths of the antibodies used for flow cytometry. AF, Alexa Fluor; APC, allophycocyanin; BV, brilliant violet; FITC, fluorescein isothiocyanate; PE, phycoerythrin; PerCP, peridinin-chlorophyll protein complex. [file 13287_2020_1663_MOESM1_ESM.pptx]

## Slide 1
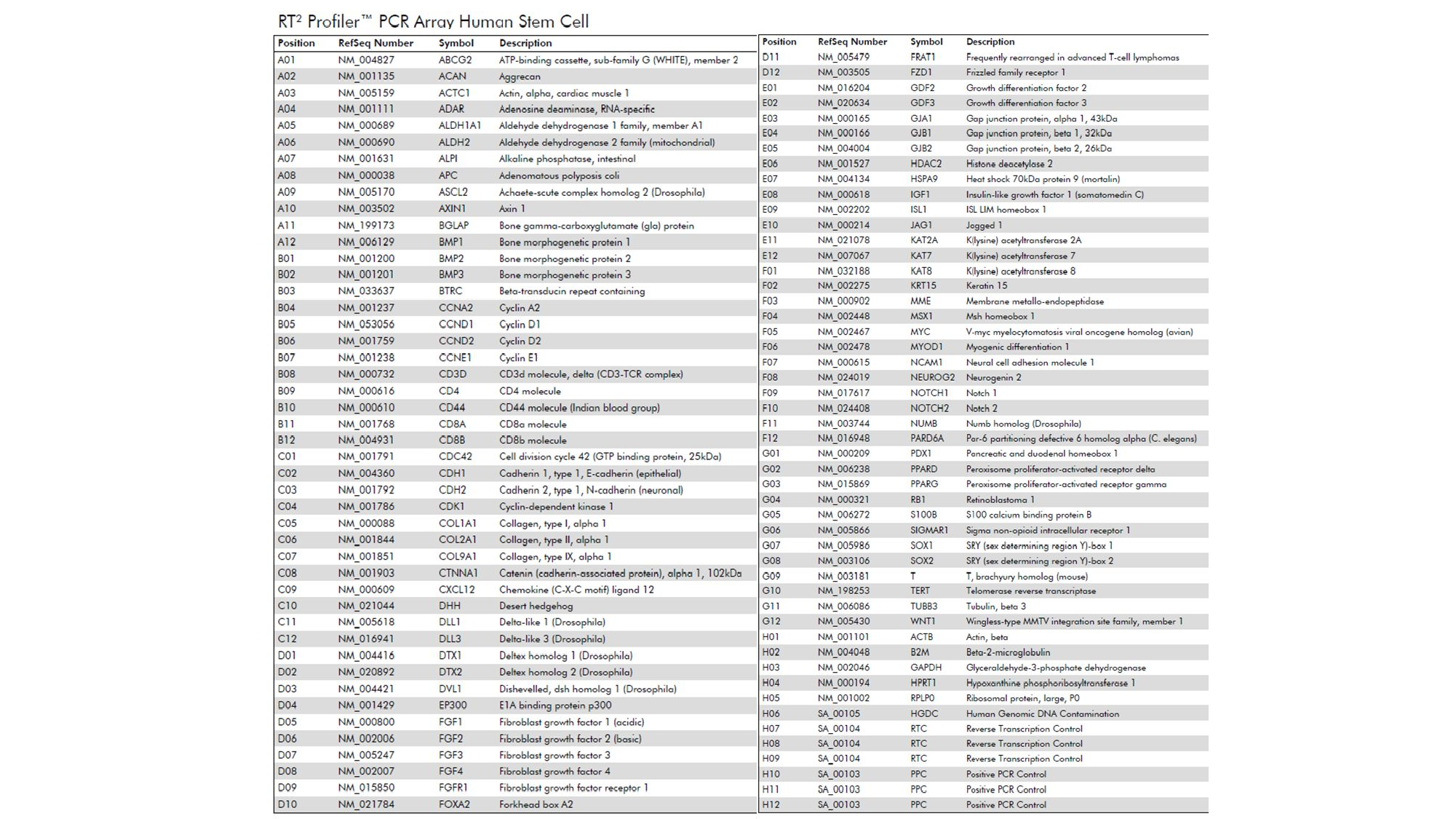

Supplement: Supplementary file 2 — Additional file 2 : Supplementary Figure S1. Detailed information on the contents of the RT2 Profiler™ Human Stem Cell PCR Array as supplied by Qiagen, including gene description and position in array. [file 13287_2020_1663_MOESM2_ESM.pptx]
